# Knowledge Acquisition and Social Support in Online Health Communities: Analysis of an Online Ovarian Cancer Community

**DOI:** 10.2196/39643

**Published:** 2022-09-13

**Authors:** Yu Chi, Khushboo Thaker, Daqing He, Vivian Hui, Heidi Donovan, Peter Brusilovsky, Young Ji Lee

**Affiliations:** 1 School of Information Science University of Kentucky Lexington, KY United States; 2 School of Computing and Information University of Pittsburgh Pittsburgh, PA United States; 3 School of Nursing University of Pittsburgh Pittsburgh, PA United States; 4 Department of Biomedical Informatics University of Pittsburgh Pittsburgh, PA United States

**Keywords:** online health community, ovarian cancer, health information needs, social support, knowledge acquisition

## Abstract

**Background:**

Patients and caregivers widely use online health communities (OHCs) to acquire knowledge from peers. Questions posed in OHCs reflect participants’ learning objectives and differ in their level of cognitive complexity. However, little is known about the topics and levels of participants’ learning objectives and the corresponding support they receive from members of OHCs.

**Objective:**

This study aimed to investigate the knowledge acquisition of patients and caregivers in an OHC. Specifically, we investigated the distribution and topics of posts with learning objectives at different cognitive complexity levels, the type and amount of social support provided to meet users’ learning objectives at different cognitive complexity levels, and the influence of social support on the change in learning objectives.

**Methods:**

We collected 10 years of discussion threads from one of the most active ovarian cancer (OvCa) OHCs. A mixed methods approach was used, including qualitative content analysis and quantitative statistical analysis. Initial posts with questions were manually classified into 1 of the 3 learning objectives with increasing cognitive complexity levels, from low to high, based on the Anderson and Krathwohl taxonomy: *understand*, *analyze*, and *evaluate*. Manual content analysis and automatic classification models were used to identify the types of social support in the comments, including emotional support and 5 types of informational support: *advice*, *referral*, *act*, *personal experience*, and *opinion*.

**Results:**

The original data set contained 909 initial posts and 14,816 comments, and the final data set for the analysis contained 560 posts with questions and 3998 comments. Our results showed that patients with OvCa and their caregivers mainly used OHCs to acquire knowledge for low- to medium-level learning objectives. Of the questions, 82.3% (461/560) were either *understand*- or *analyze*-level questions, in which users were seeking to learn basic facts and medical concepts or draw connections among different situations and conditions. Only 17.7% (99/560) of the questions were at the *evaluate* level, in which users asked other OHC members to help them make decisions or judgments. Notably, OvCa treatment was the most popular topic of interest among all the questions, regardless of the level of learning objectives. Regarding the social support received for different levels of learning objectives, significant differences were found in the *advice* (*F*_2437.84_=9.69; *P*<.001), *opinion* (*F*_2418.18_=11.56; *P*<.001), and *emotional support* (*F*_2395.88_=3.24; *P*=.01), as determined by one-way ANOVA, whereby questions at the *evaluate* level were more likely to receive *advice*, *opinion*, and *emotional support* than questions at the lower levels. Additionally, receiving social support tends to drive users to increase the cognitive complexity of the learning objective in the next post.

**Conclusions:**

Our study establishes that OHCs are promising resources for acquiring knowledge of OvCa. Our findings have implications for designing better OHCs that serve the growing OvCa community.

## Introduction

### Background

Online health communities (OHCs), also known as online support groups, are 1 of the 3 primary channels for health consumers seeking health information on the web in addition to search engines and health professionals [1]. Numerous studies have provided substantial evidence that patients benefit from OHC participation [2-5]. OHCs facilitate information exchange and knowledge acquisition among users. For people with cancer and their caregivers, who have a constant and evolving need for information, OHCs are particularly important for around-the-clock availability, immediate and asynchronous communication, and anonymity [6,7].

**Figure 1:**
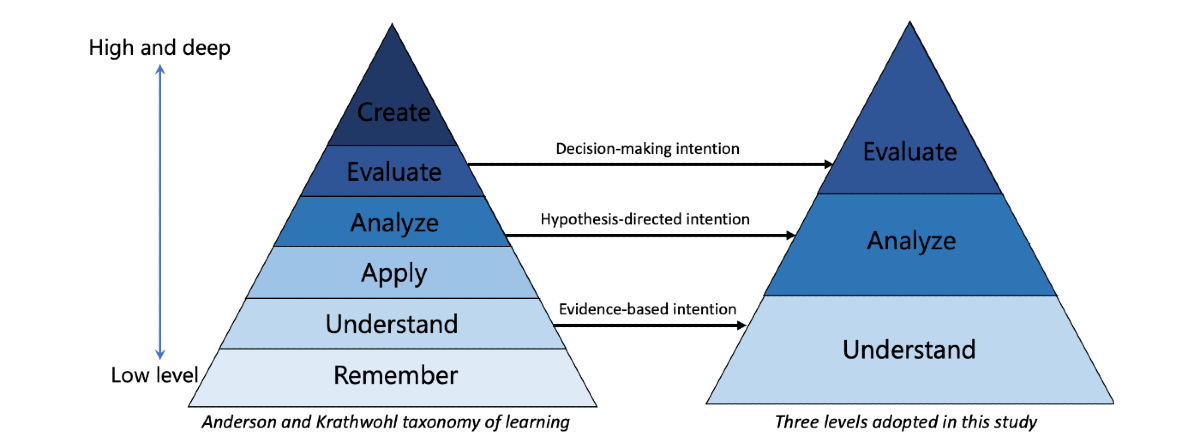
Adapted from the Anderson and Krathwohl taxonomy of learning [8].

Users ask questions on OHCs for knowledge acquisition. Questions posed by patients to acquire knowledge to meet their learning objectives vary in cognitive complexity. The cognitive complexity of learning objectives describes the cognitive skills and abilities the learner desires to achieve. For example, a question seeking advice on treatment decisions from peers (eg, surgery vs biological therapies) is cognitively more complex than one looking for facts in medical directions (eg, how many times a day is a pill to be taken). To identify the cognitive complexity level of learning objectives in OHC users’ questions, this study borrowed the Anderson and Krathwohl taxonomy of learning (A&K taxonomy) [8] from educational psychology. This taxonomy was first proposed by Bloom in 1956 [9] and later revised by Anderson and Krathwohl [8]. As shown in Figure 1, the A&K taxonomy defines 6 levels of learning objectives with increasing cognitive complexity. From low to high (ie, cognitively simple to complex), the 6 levels are *remember*, *understand*, *apply*, *analyze*, *evaluate*, and *create*. The theory assumes that to achieve a higher level of learning objectives, one must master the lower levels.

This study chose 3 levels, *understand*, *analyze*, and *evaluate*, rather than adopting all 6 levels because they are close to real web-based health information–seeking scenarios. As found in the analysis by Cartright et al [10], of queries from web search engines, there are 2 representative web-based health information–seeking intentions: evidence based and hypothesis directed. With the evidence-based intention, one mainly focuses on locating information regarding signs and symptoms, which can be mapped to the *understand* level of learning. The hypothesis-directed intention, which drives individuals to draw connections and discriminate among different uncertain situations and conditions, aligns with the *analyze* level. Finally, the *evaluate* level corresponds to the decision-making intention, which involves seeking information to make a treatment decision.

Reciprocity is another substantial benefit of OHCs [11,12]. Knowledge building and collaborative knowledge production take place through discourse among members of OHCs [13]. Peer users of the community, who usually face the same health condition and endure a similar experience, can provide social support by replying to the initial questions and follow-up discourse [3,14]. We focus on the 2 most frequently exchanged types of social support in OHCs: informational support (ie, offers information, such as the course of the condition, treatment, finance, and insurance) and emotional support (ie, expresses emotions such as caring and concern) [5,6,15].

### Objectives

Because OHCs are a promising learning resource for patients and caregivers, an in-depth study of users’ learning objectives and the corresponding support they receive is needed. First, it must be examined whether patients and caregivers use OHCs to achieve cognitively complex or simple learning objectives. Topics and health conditions discussed in OHCs may affect the patients’ learning objectives. Savolainen [16] found that >70% of the questions in OHCs for depression sought an opinion or evaluation of an issue, resembling a high-level learning objective, whereas contrasting results were found in an OHC for alcoholism, where approximately 50% of the posts looked for factual information that serves low-level learning objectives [17]. However, there is scarce literature regarding the learning objectives of users of OHCs for cancers. To deal with the numerous physical and psychosocial consequences of survival, patients with cancer and their caregivers have been using OHCs to address various cancer-related information needs and gain knowledge about cancer [18-20]. An examination of the learning objectives of people with cancer will add to the empirical knowledge on how OHCs facilitate knowledge acquisition for patients with different health conditions.

Second, it is unclear whether all levels of learning objectives are well supported in OHCs. Higher levels of learning objectives (eg, *evaluate*) are more difficult to achieve than lower levels of learning objectives (eg, *understand*) and require support from skilled and knowledgeable peers [17,21]. In this study, we examined the type and amount of support for different levels of learning objectives by measuring the corresponding social support qualitatively and quantitatively.

Third, we are interested in investigating how users’ learning objectives change during their participation in an OHC. Moreover, if one’s learning objective is well supported by peers in the OHC, will this drive them to modify their learning objective to ask a more cognitively complex question in the OHC? The answers to these questions will shed light on the effectiveness of OHCs and the designing of OHCs as web-based learning resources.

Therefore, this paper seeks to answer the following research questions (RQs):

- RQ1: What are the distributions and topics of posts at different levels of learning objectives?
- RQ2: What type and amount of social support are provided to posts at different levels of learning objectives?
- RQ3: How do users’ learning objectives change during their participation in an OHC? Is the change in the learning objectives of users associated with the type and amount of social support received?

To answer these RQs, we collected 10 years of discussion threads from an OHC for patients with ovarian cancer (OvCa) and caregivers. Because OvCa is a rare cancer [22], health information seeking on the internet can be particularly challenging because of information scarcity and limited public awareness. In addition, OvCa is the deadliest cancer among women [22]. The 5-year relative survival rate of patients with OvCa from 2011 to 2017 in the United States was 49.1% [23]. For individuals with OvCa and their families, managing this cancer can be stressful because of intensive treatments and high rates of disease progression [24]. Owing to limitations in early detection, OvCa is often diagnosed at late stages when the likelihood of cure is low. In the United States, it is the most common cause of death due to gynecological malignancies [25]. People with OvCa use OHCs to address their OvCa-specific, treatment-related, and coping-related information needs [19]. However, owing to a lack of disease awareness, 69% of the patients with OvCa had not heard of or knew nothing about OvCa before their diagnosis, thus making the knowledge acquisition and learning process extremely difficult [26]. Furthermore, studies of people living with OvCa are relatively limited, although people with OvCa need a lot of support. There is a dearth of research investigating what information individuals with OvCa who use OHCs wish to acquire and what support they receive. The findings of this study also contribute to the knowledge on how to better support the OvCa community.

## Methods

### Research Setting: National Ovarian Cancer Coalition CancerConnect Community

We collected data from CancerConnect, an OHC for patients with OvCa, managed by the National Ovarian Cancer Coalition (NOCC). NOCC is a nonprofit OvCa advocacy organization that has devoted itself to educating and supporting patients with OvCa, survivors, and caregivers since its inception in 1991. The NOCC CancerConnect Community is one of the most active OvCa OHCs [27]. It is a peer-supported OHC with the goal of providing an open-access platform that encourages and enhances interpersonal learning via informational and emotional peer interactions. To this end, NOCC allows registered users to participate and contribute to the community in several ways, such as initiating and replying to posts, searching and reading posts and comments, creating profiles, joining groups, and sending and receiving private messages.

### Ethical Considerations

This study was reviewed and approved by the Institutional Research Board of University of Pittsburgh (STUDY20040102). In addition, permission was obtained from NOCC to conduct this study.

### Data Analysis

Our NOCC data set contained 909 OvCa discussion threads posted between June 2010 and December 2020. Each thread was made of 1 initial post and corresponding comments if any. In total, there were 909 initial posts and 14,816 comments.

**Figure 2:**
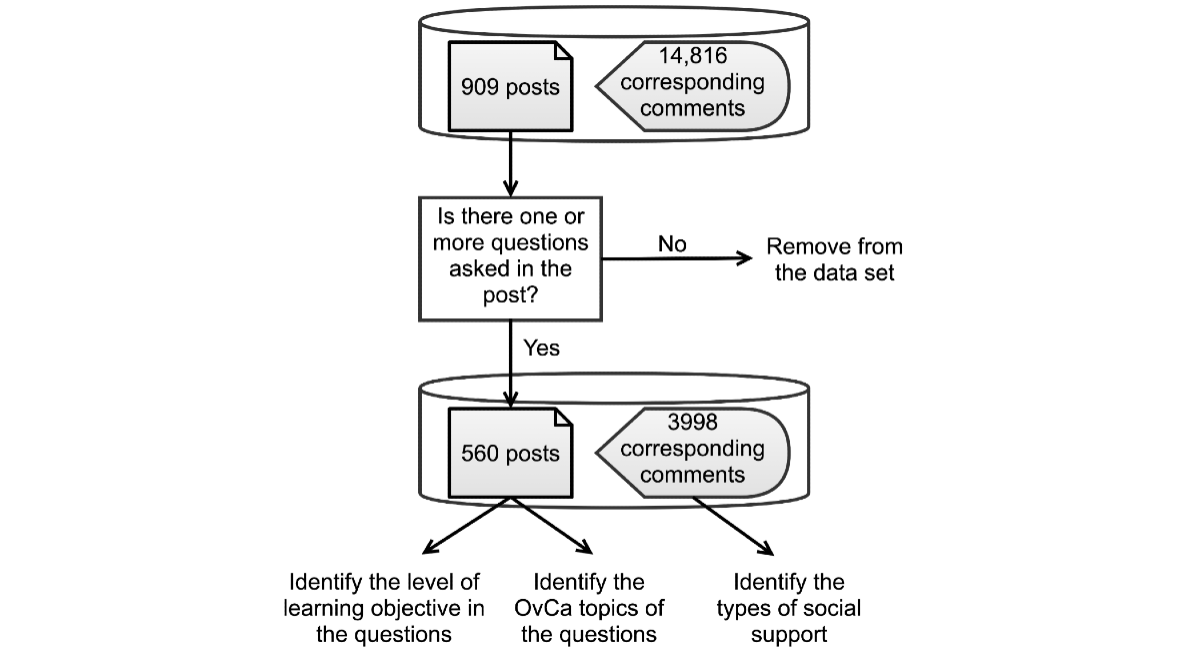
Data analysis process. OvCa: ovarian cancer.

Figure 2 illustrates the overall data analysis process. We first performed manual annotations on the 909 initial posts to determine whether there was a question articulated in the post. As a result, 560 posts and their 3998 comments were retained for further analysis. The posts without any questions mainly consisted of sharing personal updates, sharing resources, provoking discussions, and providing inspiration. The posts were then coded in terms of the level of the learning objective and OvCa-related topics. For the 3998 comments on the initial posts, we first performed manual annotation on 500 randomly chosen comments to identify the types of social support. Automatic classification models were then trained and applied to predict different types of social support in the remaining comments.

### Identifying the Level of Learning Objective

**Figure 3:**
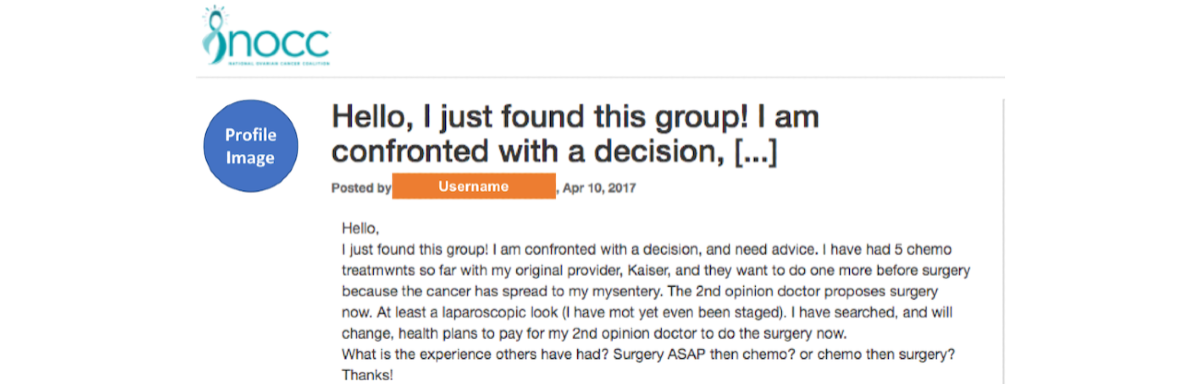
An example of an evaluate-level question.

As mentioned earlier, we borrowed 3 levels from the A&K taxonomy of learning [8] to identify the level of learning objectives in the users’ questions. The descriptions of each level of the learning objective and the deidentified example questions are displayed in Table 1. To achieve higher levels in the A&K taxonomy, one must master the lower levels in the hierarchy. Therefore, the 3 levels of learning objectives were coded mutually exclusively. For example, Figure 3 shows a post with the *evaluate* level of learning objective, as the poster described her situation and sought decision-related information from peers. The real username and user profile image are removed for privacy.

Two coders (YC and KT) applied the coding framework to 100 sample posts to determine the level of the learning objective that best describes the cognitive complexity of the questions. Substantial agreement was achieved between the 2 coders on the 100 sample posts (percentage agreement=0.79; Cohen *κ*=0.72), indicating an acceptable level of agreement [28,29]. The 2 coders then met to discuss any disagreements. Throughout the discussion, all disagreements were addressed, and no changes were made to the codebook. A coder annotated the remaining posts by using the codebook.

**Table 1:** Coding framework of learning objective in the initial post.

Learning objective	Description	Example question
Understand	Pursuit of facts, concepts, and ideas by describing, explaining, identifying, detailing, interpreting, summarizing, and so on	“Hi does anyone have information on AMG 386? Thank You”
Analyze	Pursuit of connections and relationships among multiple concepts by differentiating, comparing, distinguishing, contrasting, sorting, and so on	“I recently developed small red dots all over my legs, look like little blood marks. I’m on Avastin and wonder if anyone has experienced these marks on their body?”
Evaluate	Pursuit of decision or judgment given specific conditions by appraising, arguing, judging, selecting, critiquing, weighing, recommending, assessing, predicting, and so on	“Hi Sisters, I finished front line 12/8, and ca has be tested 3 times since. The last one showed 2 point increase and Dr wasn’t concerned as said basically save number 28 to 30. This was 1/22. Today it has went up .8. Any reason to be concerned since trend is upward? I’m concerned of this continuing and I’m already full of worry.”

### Identifying the OvCa Topics of Questions

To better understand OvCa users’ information needs at different levels of learning objectives, the topics of the questions in the initial posts were annotated through content analysis. The coding framework was inductively developed by a nurse practitioner by immersing herself in the posts. A coding framework with 13 topics was established initially.

Using this framework, the 2 coders individually annotated all the posts. Questions in each post included 1 or multiple topics. Later, topics that appeared in <10 posts were further grouped into *Others*. Consequently, 9 codes were used to classify the topics of information needs in the initial posts (Table 2). An acceptable interrater agreement was obtained between the 2 coders, with an average percentage agreement of 0.94 and Cohen *κ* coefficient of 0.72, ranging from 0.62 to 0.81 across 9 categories [28,29]. The 2 coders discussed and resolved all disagreements and reached an agreement in all cases.

**Table 2:** Coding framework of topics of questions.

Topic	Description	Code
Disease management	Information needs related to ovarian cancer disease management, such as diagnosis, prognosis, finding gynecologic oncologist, preparing for visit, advance care planning or advance directives, borderline malignant tumors, prophylactic surgery, secondary prevention, monitoring for recurrence, management of recurrence, and supportive care or palliative care	DM^a^
Symptom management	Information needs related to ovarian cancer symptom management, such as fatigue, sleep, bowel, pain, neuropathy, cognitive memory, nausea, vomiting, bloating, ascites, appetite, appearance, shortness of breath, lymphedema, urinary, early menopause, ostomy management, rash, anemia, mouth sore, and myelosuppression	SM^b^
Treatment	Information needs related to ovarian cancer treatment, such as medications, surgery, radiation, chemotherapy, biological therapies, and clinical trials	TM^c^
Treatment decision	Information needs related to ovarian cancer decision-making, such as how to make treatment decisions	TD^d^
Emotional management	Information needs related to emotional management, such as anxiety, depression, fear of recurrence, mood swings, coping, grief, and loss	EM^e^
Self-management	Information needs related to self-management, such as nutrition, spiritual support, physical activity, and relationship with loved ones	SF^f^
Practical needs	Information needs related to practical needs, such as finance, insurance, employment, legal, and community resources	PN^g^
Caregiving	Information needs related to caregivers’ needs, such as stress, caregiver coping, grief, and loss	CG^h^
Others	Other ovarian cancer–related information needs, such as communication, sexuality, rehabilitation, complementary therapy and integrative medicine, ovarian cancer organization, and facilities	OT^i^

^a^DM: disease management.

^b^SM: symptom management.

^c^TM: treatment.

^d^TD: treatment decision.

^e^EM: emotional management.

^f^SF: self-management.

^g^PN: practical needs.

^h^CG: caregiving.

^i^OT: others.

### Identifying the Types of Social Support

The 2 most common types of social support exchanged in OHC are informational and emotional support [5,6]. In this study, as the aim was to investigate what information users receive as answers to their questions, the informational support provided in the comment was further classified by using the framework proposed by Chuang and Yang [17]. Chuang and Yang [17] identified five types of informational support:

1. Advice: the comment offers ideas, suggestions, and actions to cope with challenges.
2. Referral: the comment refers to information sources such as books, websites, and contacts.
3. Fact: the comment offers facts or reassesses the situation.
4. Personal experience: the comment shares personal stories or incidents.
5. Opinion: the comment offers a view or judgment about something. However, this is not necessarily based on facts or knowledge.

In addition, emotional support was marked if a comment provided empathy, encouragement, or appreciation [12].

**Figure 4:**
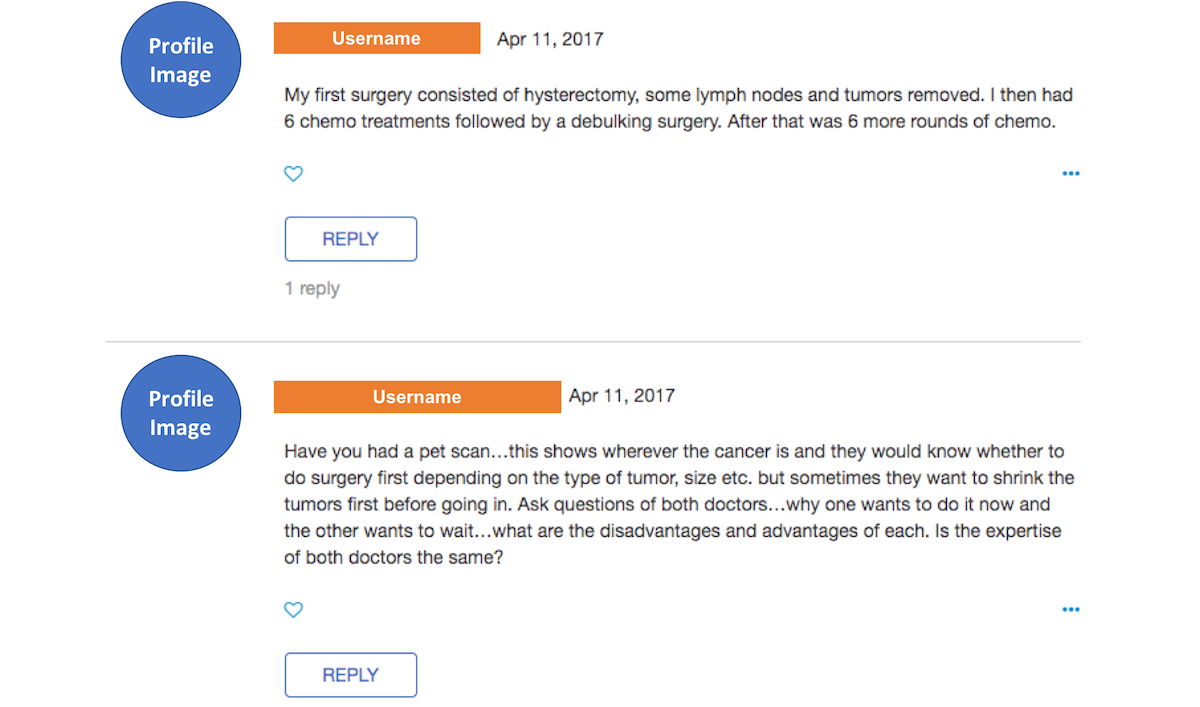
Examples of comments.

All 6 types of social support, including emotional support and 5 types of informational support, were coded in a binary fashion, and a comment could provide 0, 1, or multiple types of support. If no informational or emotional support could be identified, the comment was coded as “Others.” For example, Figure 4 displays 2 comment examples that replied to posts shown in Figure 3. The first comment was coded as “1” for providing a *fact* and “0” for all other types of informational and emotional support. The second comment was coded as “1” for providing a *fact* and an *advice* and “0” for all others.

The social support types provided in the 3998 comments were identified in 3 steps. First, 2 coders coded 150 sample comments to ensure the reliability of the coding framework. On average, an agreement rate with percentage agreement of 0.94 and Cohen *κ* of 0.84 were achieved, indicating an almost perfect agreement [28,29]. Second, after addressing all disagreements, a coder coded 350 more comments. As a result, a data set of 500 comments was obtained, in which each comment contained a comment text and corresponding support labels. Third, as it would be impractical to annotate all 3998 comments, the decision was made to build machine learning–based classifiers by using the already annotated comments. In total, 6 machine classifiers were built for each support type. A pretrained Bidirectional Encoder Representations from Transformers (BERT) language model [30] was fine-tuned for each classification task. BERT was used because it obtained good classification accuracy with less data on different downstream text classification tasks, such as sentiment and emotion classification [30]. The data set was split into 3 folds with a 70:10:20 ratio for training, validation, and testing, respectively. The accuracy reported in Table 3 is based on the testing fold. The interrater agreement between the 2 coders and performance of the classification models are presented in Table 3. The code for the model and access to our model are listed on GitHub [31]. Finally, the models were applied to predict the social support types for the remaining comments.

**Table 3:** Interrater agreement between human annotators and classification score for social support types in the comments.

Support type	Interrater agreement		Support type prediction		
	Percentage agreement	Cohen *κ*	Precision	Recall	*F*-score
Advice	0.96	0.88	0.77	0.85	0.81
Referral	0.98	0.94	0.82	1.00	0.90
Fact	0.93	0.86	0.82	0.77	0.79
Personal experience	0.90	0.80	0.95	0.87	0.91
Opinion	0.93	0.79	0.81	0.81	0.81
Emotional support	0.91	0.82	0.91	0.74	0.82
Others	0.95	0.76	N/A^a^	N/A	N/A
Average	0.94	0.84	0.85	0.84	0.84

^a^N/A: not applicable.

## Results

### Overview

Of 909 initial posts, 560 (61.6%) were associated with learning objectives, as indicated by the questions asked in the posts. The following results were based on the analysis of the 560 initial posts with identified learning objectives and 3642 comments that provided at least one type of support.

### Learning Objectives in the Initial Posts (RQ1)

#### Distribution of Users’ Learning Objectives in the Initial Posts

Among the 560 posts with questions, the *analyze* objective was the most common, accounting for almost half of the total (257/560, 45.9%). Following this, 36.4% (204/560) of the posts with questions sought *understand*-level knowledge, whereas *evaluate*, the most complex learning objective, only accounted for 17.7% (99/560) of the question-asking posts. This result suggests that people with OvCa mainly use the NOCC community to look for simple knowledge, such as facts, concepts, or relationships between facts and concepts, rather than complex knowledge relating to treatment decisions and judgments.

#### Number of Topics

In most of the initial posts, users tended to seek information and knowledge about 1 (363/560, 64.8%) or 2 (176/560, 31.4%) topics per post. There were only 21 posts in which users consulted their peers on >2 OvCa topics (21/560, 3.8%).

The initial posts were grouped according to the 3 levels of learning objectives; the average number of topics in each group is presented in Table 4. A one-way between-subject ANOVA was performed on the number of topics in 1 post as a function of the level of learning objective. With violation of the assumption of homogeneity of variance, an *F*-test with Brown-Forsythe adjustment was conducted. The results suggested a statistically significant difference in the number of topics among the different levels of learning objectives (*F*_2193.364_=72.54; *P*<.001). A Games-Howell post hoc test revealed that there were significantly more topics in the posts asking for an *evaluate*-level learning question (N=1.83; *P*<.001) than in posts with the *analyze*-level learning objective (N=1.50; *P*<.001). The posts seeking *understand*-level knowledge consisted of the least number of topics compared with the 2 higher levels (N=1.05; *P*<.001). The difference in the number of topics may indicate that people with OvCa tend to acquire information across multiple topics to obtain *evaluate*-level knowledge. By contrast, for lower-level learning objectives, their information needs were more likely to focus on 1 specific topic.

**Table 4:** Number of topics per post at each level of learning objective.

Learning objective	Topics per post, mean (SD)	Posts, n (%)
Understand	1.05 (0.24)	204 (36.4)
Analyze	1.50 (0.54)	257 (45.9)
Evaluate	1.83 (0.73)	99 (17.7)
Total	1.40 (0.57)	560 (100)

#### Category of Topics

Using the coding framework in Table 2, the questions in the initial posts were classified into 9 categories based on OvCa-related topics. In this section, 2 results for the topic categories are presented. First, topics were grouped by different levels of learning objectives to show what OvCa-related knowledge patients and caregivers wanted to acquire. Then, for posts with >1 topic, the frequencies of all topic pairs were examined to further demonstrate what topics tended to be inquired about together.

**Figure 5:**
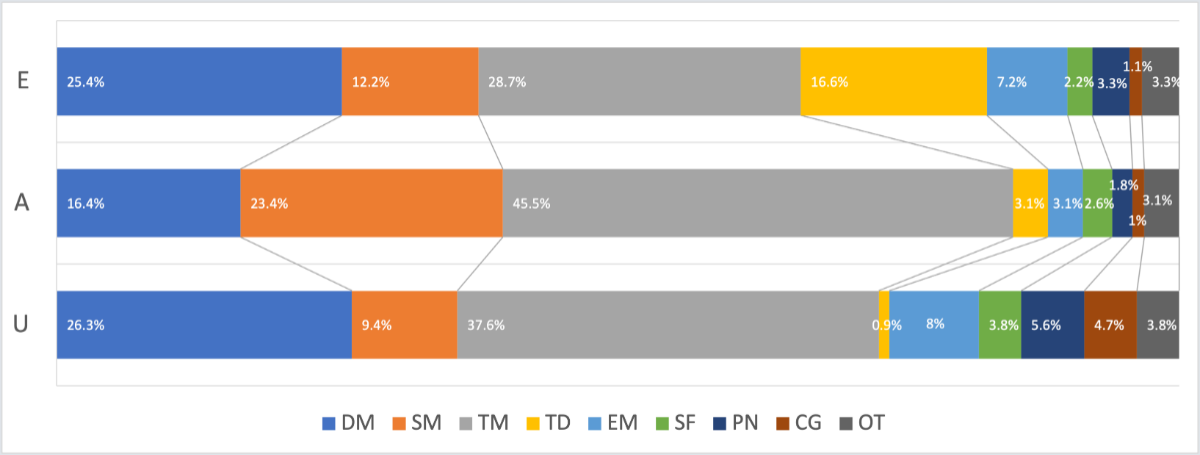
Distribution of ovarian cancer topics at each learning objective level. A: analyze; CG: caregiving; DM: disease management; E: evaluate; EM: emotional management; OT: others; PN: practical needs; SF: self-management; SM: symptom management; TD: treatment decision; TM: treatment; U: understand.

Figure 5 shows the distribution of the 9 OvCa-related topics at each level of learning objective. Each bar represents the posts of 1 of the 3 levels of learning objectives, whereas segments in the bar denote the portion of a topic among all posts with the same level of learning objective. Segments of the same color were comparable.

It is evident that *treatment* is the most popular topic of interest in all knowledge acquisition posts, with a higher proportion in the *analyze* level (175/385, 45.4%) than in the other 2 levels of learning objectives. This result indicated that comparing or differentiating treatment information was a common need among people with OvCa in OHCs. In addition, pursuing treatment information to understand or evaluate was frequent, which might be because the treatment information of OvCa was complex and scattered, making the topic of treatment the dominant information needed across all the learning objectives. *Analyzing symptom management* is the second most prevalent information needed, whereas understanding and evaluating symptom management information is not that popular. The results suggest that for symptom management, patients and caregivers struggle more with the differentiation or connection among different symptoms than with learning about basic symptoms or making judgments.

On the contrary, *disease management* was more associated with the *understand* and *evaluate* levels of learning objectives than the *analyze* level, implying that people with OvCa needed support for interpreting disease information such as diagnosis, prognosis, and recurrence on both a basic fact or concept level and a higher judgment or decision level. It is notable that *treatment decisions* accounted for a significant portion (30/181, 16.6%) of the *evaluate* level. However, it is questionable whether users should use OHC as a resource for making treatment-related decisions. *Emotional management* and *practical needs* presented similar patterns: the proportions of *understand* and *evaluate* questions were higher than *that of analyze* questions. Caregiving information accounted for a much greater share of *understand* questions than the other two. Finally, the ratios of the other topics were similar for all 3 levels of learning objectives.

Chi-square results revealed a significant association between the levels of learning objectives and the topics of *disease management* (*χ*^2^_2_=17.2; *P*<.001), *symptom management* (*χ*^2^_2_=40.2; *P*<.001), *treatment* (*χ*^2^_2_=38.6; *P*<.001), *treatment decision* (*χ*^2^_2_=85.8; *P*<.001), and *emotional management* (*χ*^2^_2_=7.7; *P*=.02). However, no significant association was found between the learning objective levels and topics of *self-management* (*χ*^2^_2_=0.0; *P*=.99), *practical needs* (*χ*^2^_2_=0.3; *P*=.19), *caregiving* (*χ*^2^_2_=0.4; *P*=.09), and others (*χ*^2^_2_=0.6; *P*=.71).

**Figure 6:**
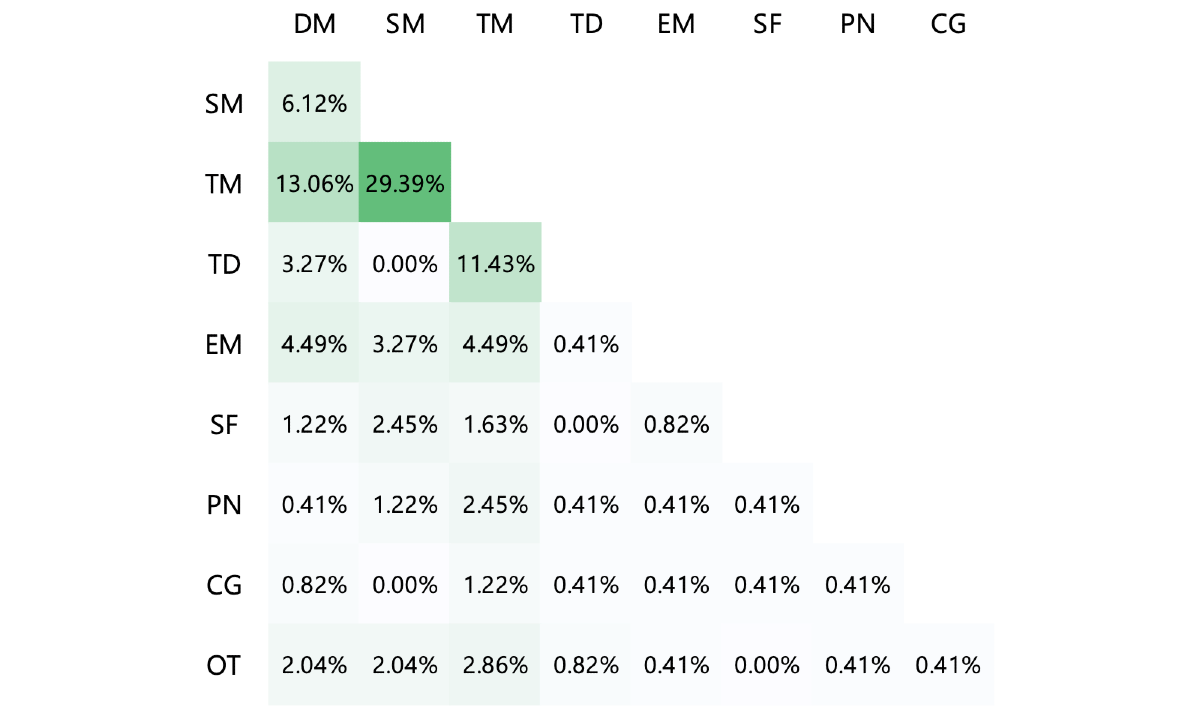
Co-occurrence of topic pairs in 1 post (darker color indicates larger proportions). CG: caregiving; DM: disease management; EM: emotional management; OT: others; PN: practical needs; SF: self-management; SM: symptom management; TD: treatment decision; TM: treatment.

Figure 6 shows the proportions of different topic pairs among the 245 topic pairs extracted from questions with >1 topic. Notably, treatment and symptom management were most likely to appear together in a single post (72/245, 29.4%). In addition, patients with OvCa and their caregivers tended to learn about treatment along with disease management or treatment decisions.

### Social Support in the Comments (RQ2)

#### Number of Replies to Posts at Different Levels of Learning Objectives

The 3642 comments providing support were grouped based on the learning objective in the post. Posts with the *understand* level were likely to receive the largest average number of comments from peers (N=7.68), followed by the *evaluate* (N=7.07) and *analyze* (N=5.63) levels. However, the results of the one-way ANOVA suggested no statistically significant difference between the average number of comments among the 3 levels of learning objectives (*F*_2451.295_=2.712; *P*=.07).

#### Social Support Provided for Posts at Different Levels of Learning Objectives

**Figure 7:**
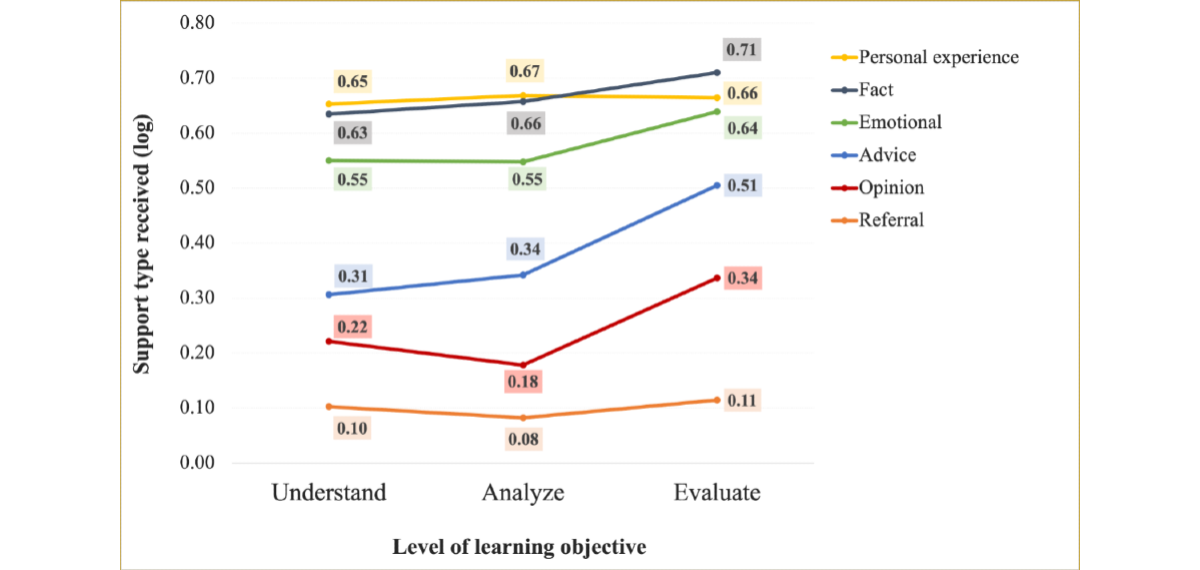
Type and amount of social support provided for questions at each learning objective level.

The types and amount of social support provided by the repliers in each comment were aggregated by posts. Figure 7 shows the number of different types of support received in each post belonging to each learning objective. Log transformation is applied to the total number of each type of comment and plotted in the line chart. In general, the largest number of supportive replies was provided to posts with the evaluate-level learning objective, followed by the *understand* level, and it was the least for the *analyze*-level learning objective.

As determined by one-way ANOVA, significant differences among the 3 levels of learning objectives were found in *advice* (*F*_2437.84_=9.69; *P*<.001), *opinion* (*F*_2418.18_=11.56; *P*<.001), and *emotional support* (*F*_2395.88_=3.24; *P*=.01) levels. A Games-Howell post hoc test revealed that posts seeking *analyze*-level knowledge received significantly less *opinion* support compared with *understand*-level (*P*=.002) and *evaluate*-level posts (*P*<.001). The amount of *advice* support at the *evaluate* level was significantly higher than that at the *analyze* (*P*<.001) and *understand* (*P*=.001) levels. For *emotional* *support*, a significant result was found only between *analyze* and *evaluate* (*P*=.02) levels.

### Influence of Social Support on Change in the Learning Objective (RQ3)

#### Overview

Because some users posted >1 posts with learning objectives in NOCC, this allowed the researcher to unveil how the learning objectives of the same user change over time. In total, 344 distinct users posted 560 posts with learning objectives. Most users (244/344, 70.9%) posted only 1 post, and 29.1% (100/344) of users posted multiple posts. Among the 100 users who posted >1 posts with learning objectives, 60, 17, 9, and 14 posted 2, 3, 4, and >5 posts, respectively, with learning objectives. These 100 users were further examined to uncover changes in their learning objectives in the NOCC and the influence of social support on the change.

The change in the learning objective is defined as the transition between the level of the learning objective in post *P_i_* and post *P_i+1_* for the same user *U*. The change in learning objectives was classified into 3 categories based on the transition from post *P_i_* to *P_i+1_*: knowledge increase, knowledge decrease, and no change. For example, if a user posted 3 initial posts (ie, *P_1_*, *P_2_*, and *P_3_*) in the NOCC forum and the level of learning objective in them are *P_1_*—*understand*, *P_2_*—*analyze*, and *P_3_*—*analyze*, then the change in learning objective from *P_1_* to *P_2_* is knowledge increase, and the change from *P_2_* to *P_3_* is no change. In total, 216 changes in learning objectives were identified from the 100 users who contributed multiple posts in the NOCC forum.

#### Change of Learning Objectives of the Same User

In general, 41.7% (90/216) of the pairs of 2 consecutive posts sought information on the same level of learning objectives, which resulted in *no change*. *Knowledge increase*, in which the learning objective in the subsequent post was higher than the previous one, was the second most frequent (70/216, 32.4%). The least frequent type of change was *knowledge decrease* (56/216, 25.9%). It can be inferred that NOCC users were more likely to increase or remain at the same level of learning objectives as they continued posting, asking questions, and acquiring knowledge in the same forum.

We also examined the specific types of transitions from different levels of learning objectives (eg, from *understand* to *understand*). This helped reveal how the current level of learning objective affected the subsequent post’s learning objective. First, from *analyze* to *analyze* (A→A: 57/216, 26.4%) was the most common transition. The amount and ratio are also higher than those from *analyze* to *understand* (A→U: 22/216, 10.2%) and *analyze* to *evaluate* (A→E: 24/216, 11.2%), suggesting that *analyze*-level questions were likely to be followed by another *analyze*-level question than the increase or decrease in levels of learning objectives of the same user. Second, after asking an *understand*-level question, users tended to increase the level of learning objective and ask an *analyze*-level question (U→A: 36/216, 16.7%). This possibility is higher than asking another *understand*-level question (U→U: 27/216, 12.5%) or *evaluate*-level question (U→E: 10/216, 4.6%). This might be attributed to the fact that the *understand*-level learning objective was relatively easy to achieve, or the users’ OvCa-related knowledge might evolve and increase over time, driving them to pursue a higher level of learning. Third, *evaluate*-level posts were mainly followed by *analyze*-level posts (E→A: 22/216, 10.2%) or *understand*-level posts (E→U: 12/216, 5.6%). Only rarely would users ask another *evaluate*-level question (E→E: 6/216, 2.8%). In addition, users were more likely to increase the learning objective by 1 level (ie, U→A: A→E) or decrease it by 1 level (ie, E→A: A→U) in 2 consecutive posts than to increase or decrease it by 2 levels (ie, U→E: E→U), indicating that the change in learning objectives was a gradually evolving process.

#### Social Support Received and Change of Learning Objective

**Figure 8:**
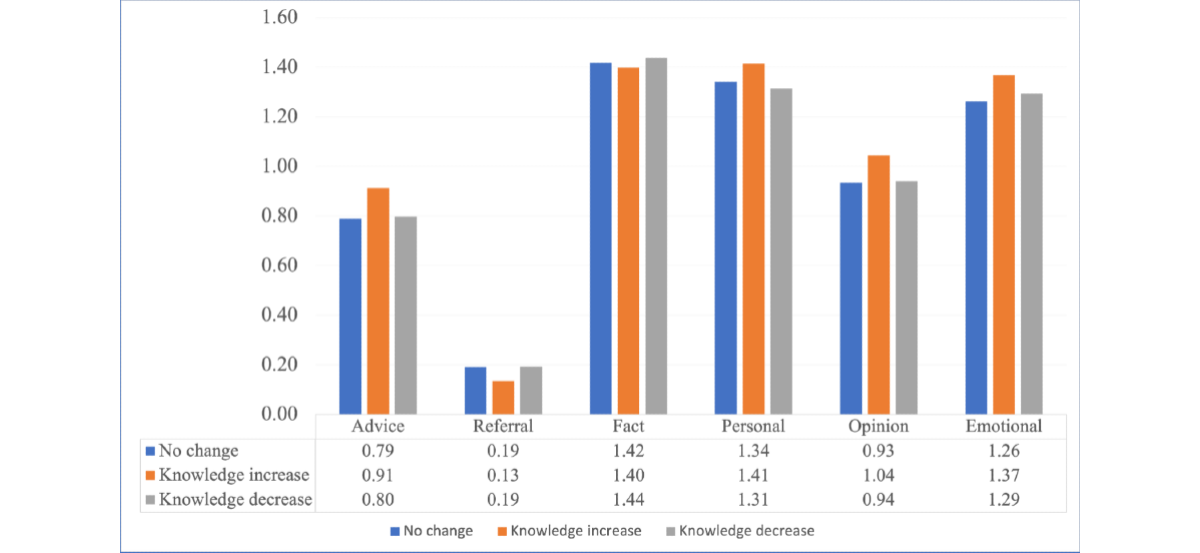
Amount and type of social support received and change in the learning objective level.

Figure 8 shows how the type and amount of social support received for the current post influenced users’ learning objectives in the next post. On average, for most types of social support, when users received more support, including *advice*, *personal experience*, *opinion*, and *emotional support*, they were more likely to increase their learning objective in the next post, rather than decrease or maintain the same level of learning objective. No statistically significant differences were found between the 3 types of changes.

## Discussion

### Overview

This study investigated knowledge acquisition by people with OvCa in an OHC. We borrowed three levels of learning objectives from the A&K taxonomy: *understand*, *analyze*, and *evaluate*. The results revealed (1) the distributions and topics of posts at different learning objective levels, (2) the type and amount of corresponding social support at each level, and (3) the influence of social support on changes in learning objectives. The principal findings, contributions, implications, and limitations of this study are discussed in the following sections.

### Principal Findings

Our results showed that NOCC was mainly used by patients with OvCa and their caregivers to address information needs with low- to middle-level learning objectives. Of the questions, 82.3% (461/560) were either at the *understand* or *analyze* levels of cognitive complexity, in which the user initiates a post to pursue basic facts and concepts or connections and relationships among multiple concepts. Notably, only 17.7% (99/560) of the posts with questions were associated with an *evaluate*-level learning objective, in which the users asked other OHC members to help them make decisions or judgments based on their specific conditions. These results are partially different from the findings in [16], where >70% of the posted questions in the web-based discussion forums sought an opinion or evaluation of an issue, resembling an *evaluate*- or *analyze*-level question, whereas the need for factual and procedural information was less common. These conflicting results could be attributed to the different health conditions studied. In in the study by Savolainen [16], the topic of interest in the threads was depression, whereas in this study, it was OvCa, which is listed as a type of rare cancer by the National Institutes of Health [1]. Therefore, the general public lacks disease awareness and education regarding OvCa, and the information is complex and scattered. This might cause OHC users to seek basic facts and concepts at the *understand* level more often. In addition, the prevalence of *analyze*-level questions could be explained by the fact that OvCa is a complex disease. Because the diagnosis, staging, and treatment are complex, patients and caregivers have to learn and sort out which information applies to them and which does not. For example, on average, women with OvCa under treatment need to manage 12 concurrent symptoms [32].

Regarding OvCa-related topics, treatment is the most popular topic of interest among all the information needs, regardless of the level of learning objectives. This finding is in accordance with the results in the study by Madathil et al [19], in which treatment-related information was found to be the most sought-after information by patients (41.3%) compared with OvCa-specific and coping information. Data analyses were conducted at the Ovarian Cancer National Alliance, another OHC for OvCa. We identified 9 different topics by using our fine-grained topic classification framework, and the posts were classified in a nonmutually exclusive manner. Treatment was still found to be the most popular topic. This finding further underlines the high demand for treatment-related information and support among people with OvCa. It is also noteworthy that treatment decision accounted for a large share at the *evaluate* level despite the concern that an OHC might not be an appropriate resource to ask for treatment-related decisions. Such findings add to the demand for research efforts to assess the quality of treatment-related decisions shared by peers in OHCs.

In addition, we examined the type and amount of informational support in the comments, providing a means to study the quantity and quality of information that OHC users can acquire at different levels of knowledge acquisition. In general, users in the NOCC group received the largest number of comments for *understand*-level learning objective (N=7.68), followed by evaluate-level (N=7.07) and *analyze*-level (N=5.63) learning objectives. However, the number of comments itself was not enough to reflect the quality and quantity of social support in the OHC because a comment could provide 0, 1, or multiple types of social support; therefore, we classified the types of social support in the comment, especially informational support.

Descriptive results indicated that, in general, the total amount of social support of all types was the largest for evaluate-level learning, followed by *understand*-level learning, and it was the least for *analyze*-level learning. For each type of social support, *fact* was acquired the most compared with other types of support. This result is consistent with the results in the study by Chuang et al [17], which were based on a manual analysis of an alcoholism OHC. Regarding the effect of the learning objective, the results suggest that more *advice*, *opinions*, and *emotional support* were obtained for questions seeking *evaluate*-level learning. A possible explanation for this finding is that some subjective knowledge, to a certain extent, was needed to support people with OvCa’s information needs of evaluate-level learning. As justified by the interviewees in the study by Harkin et al [2], practical advice shared by peers in OHCs was welcomed by many interviewees, as such information led them on a “journey to become informed.” It is also notable that although the questions with the *analyze*-level learning objective were the most frequently posted in the OHC, they received the smallest number of average comments and the least amount of almost all types of social support in the comments. Measures beyond the number of comments and support are required to explore this finding in the future.

Finally, we examined multiple posts from the same user, and the results demonstrated that OvCa users’ learning objectives changed during OHC use. This change was reflected by the transition from the current post’s learning objective to the subsequent post’s learning objective. Most of the users who posted >1 post with a learning objective in the NOCC tended to increase their learning objective (70/216, 32.4%) or remained at the same level of learning objective (90/216, 41.7%), as they continued posting and seeking information in the same forum. Furthermore, for users who increased their learning objective in the next post, a larger amount of support in *advice*, *personal experience*, *opinion*, and *emotional support* was observed in the current post (Figure 8). In other words, receiving more social support might drive the users to acquire higher-level knowledge in the same OHC. Although the result was not statistically significant, this finding adds to previous studies that have demonstrated the effect of social support on member retention and engagement [5,6,33] and contributes new evidence on the potential effects of social support on collaborative knowledge building and generation in web-based communities [13]. In-depth future research promises to investigate the relationship between receiving social support, especially informational support, and knowledge acquisition in OHCs.

### Contributions and Implications

As one of the first studies to investigate users’ knowledge acquisition in the context of OHCs, this study presents several contributions and implications to OHCs and the population of the OvCa community.

### Implications for OHC

First, although there is an extensive body of literature investigating OHCs, and it has been proven that patients and their caregivers would use OHCs to post questions and acquire knowledge [12,15,17], little has been done to differentiate knowledge acquisition with different levels of learning objectives and the associated social support provided by peers in OHCs. Our study contributes empirical evidence and demonstrates that user interactions in OHCs can be described and studied from a knowledge acquisition perspective. Not all information needs regarding the underlying cognitive complexity of the learning objectives are identical. Our study also demonstrated that OHC is a promising resource for users to address information needs with different cognitive complexities and that OHCs can help users to improve knowledge if their information needs are well supported with informational and emotional support from peers.

Correspondingly, OHCs ought to recognize the cognitive complexity of the user’s information needs and the underlying learning objective. Importantly, the quality and quantity of social support from peers are critical for users to address their information needs and seek higher-level knowledge. Enhancing patients’ learning objectives is important because pursuing cognitively more complex learning objectives implies higher patient activation—informed and activated patients who actively engage in health care and decision-making. Higher patient activation is associated with better health-related outcomes [34,35]. Given the result that certain types of support were associated with an increase in learning objectives, algorithms or human moderators in OHCs are expected to match the level of learning objectives in the original post with the appropriate types of social support from active peers.

With their social features, OHCs amplify the benefits of a wealth of information as well as the negative emotions shared by peers. In addition, there are concerns about the quality of the narratives shared by patients in OHCs [36,37]. False information and rumors can cause false expectations [2]. To deal with the downside of OHCs, it is suggested that the content be carefully administered by moderators with professional backgrounds. Attention should be devoted to information-seeking posts with high cognitively complex learning objectives such as pursuing judgments and decisions from peers. In addition, some high-quality learning materials can be developed and disseminated via OHCs, as they have been proven to be an active informal learning platform.

### Implications for OvCa Community

People with OvCa have exhibited constant and dynamic information needs, which changes based on the disease trajectory. Concurrently, their knowledge of the disease evolves gradually over the course of the disease trajectory. Most patients with OvCa have little to no knowledge of OvCa before their diagnosis due to a lack of disease awareness [26]. As the trajectory proceeds, they obtain information and gain knowledge through diverse sources, including OHCs [38]. However, the knowledge acquisition process could be extremely difficult because of the lack of OvCa-related knowledge, poor quality of some information available on the web, and inherent characteristics of OvCa [39]. The high prevalence of questions associated with low- to middle-level learning objectives found in this study further confirmed the public’s lack of awareness of OvCa and the community’s lack of disease knowledge.

By contrast, the findings highlighted the benefits of OHC in supporting the OvCa community. Patients with OvCa and caregivers address their assorted information needs in OHC and exchange information and emotional support in the community. In addition, the results based on the classification of OvCa-related topics provide insights into the information needs of people with OvCa, such as the high demand for treatment-related information and support. As there are multiple treatment options for OvCa, a more personalized search system will be beneficial for providing adjusted and dynamic treatment support. The findings provide implications for future health care providers, practitioners, researchers, and developers to design personalized health information systems that will enhance knowledge acquisition and satisfy the unmet needs of people with OvCa.

### Methodological and Theoretical Implications

In addition to the empirical and practical implications of this study, there are several theoretical and methodological implications. First, this study adopted a mixed methods approach, which allowed us to examine both the quality and quantity of the OvCa community’s knowledge acquisition in OHCs. Second, several coding frameworks originated from this study, such as the coding framework for OvCa-related topics and the coding framework for learning objectives. These frameworks can provide future researchers with an approach to unveil the complicated information requirements of the OvCa community.

### Limitations and Future Directions

Regardless of its strengths, this study has several limitations. First, this study was conducted on the NOCC. Although it is a popular OHC for people with OvCa, the results of this study might be biased toward the site used to collect the data. Second, the measurement of users’ learning objectives in this study was limited by the scope of the A&K taxonomy. Only 3 representative cognitive learning levels were selected. Such a design is based on the rationale explained in the Methods section, but we acknowledge that users’ learning and knowledge evolution was oversimplified. Knowledge acquisition is confined to research settings. Little is known about how much the participants learned via other information sources beyond information seeking and support within the OHC. In the future, a complementary obtrusive approach, such as a questionnaire, would help measure patients’ knowledge acquisition more comprehensively. Third, this study only captures OvCa-related topics based on the information needs of patients and caregivers. Other types of supportive care needs, such as interpersonal or intimacy and daily living needs, were not included in the analysis [40]. Finally, this study did not distinguish patients with OvCa according to their disease trajectory, given the scarce data in the NOCC. However, the literature suggests that the information needs of people with OvCa change with the disease trajectory [41,42]. It would be interesting to investigate whether there is a significant effect of disease trajectory on learning objectives and support in OHC. The answer to this question may help researchers and clinicians design interventions that better support patients with OvCa along their disease trajectory.

### Conclusions

This work is one of the first to investigate users’ participation in OHCs from a knowledge acquisition perspective through the analysis of a well-known OHC for OvCa. The results demonstrate that users use OHCs to address information needs with different levels of learning objectives, and simultaneously, they can acquire various types of information and emotional support in the comments from peers. Receiving support drives users to pursue higher levels of learning objectives. These findings contribute to improving OHC designs to support the OvCa community.

## Abbreviations

A&K taxonomy: Anderson and Krathwohl taxonomy of learning
BERT: Bidirectional Encoder Representations from Transformers
NOCC: National Ovarian Cancer Coalition
OHC: online health community
OvCa: ovarian cancer
RQ: research question
